# Synthesis, Surface Modification and Magnetic Properties Analysis of Heat-Generating Cobalt-Substituted Magnetite Nanoparticles

**DOI:** 10.3390/nano14090782

**Published:** 2024-04-30

**Authors:** Miloš Ognjanović, Marko Bošković, Hristo Kolev, Biljana Dojčinović, Sanja Vranješ-Đurić, Bratislav Antić

**Affiliations:** 1 VINČA Institute of Nuclear Sciences, National Institute of the Republic of Serbia, University of Belgrade, 11351 Belgrade, Serbia; markob@vin.bg.ac.rs (M.B.); sanjav@vin.bg.ac.rs (S.V.-Đ.); bantic@vin.bg.ac.rs (B.A.); 2 Institute of Catalysis, Bulgarian Academy of Sciences, 1113 Sofia, Bulgaria; hgkolev@ic.bas.bg; 3 Institute of Chemistry, Technology and Metallurgy, National Institute of the Republic of Serbia, University of Belgrade, 11000 Belgrade, Serbia; bmatic@chem.bg.ac.rs

**Keywords:** nanoparticles, nanomagnetism, surface, colloids, magnetic hyperthermia

## Abstract

Here, we present the results of the synthesis, surface modification, and properties analysis of magnetite-based nanoparticles, specifically Co_0.047_Fe_2.953_O_4_ (S1) and Co_0.086_Fe_2.914_O_4_ (S2). These nanoparticles were synthesized using the co-precipitation method at 80 °C for 2 h. They exhibit a single-phase nature and crystallize in a spinel-type structure (space group *Fd*$\bar{3}$*m*). Transmission electron microscopy analysis reveals that the particles are quasi-spherical in shape and approximately 11 nm in size. An observed increase in saturation magnetization, coercivity, remanence, and blocking temperature in S2 compared to S1 can be attributed to an increase in magnetocrystalline anisotropy due to the incorporation of Co ions in the crystal lattice of the parent compound (Fe_3_O_4_). The heating efficiency of the samples was determined by fitting the Box-Lucas equation to the acquired temperature curves. The calculated Specific Loss Power (*SLP*) values were 46 W/g and 23 W/g (under *H_AC_* = 200 Oe and *f* = 252 kHz) for S1 and S2, respectively. Additionally, sample S1 was coated with citric acid (Co_0.047_Fe_2.953_O_4_@CA) and poly(acrylic acid) (Co_0.047_Fe_2.953_O_4_@PAA) to obtain stable colloids for further tests for magnetic hyperthermia applications in cancer therapy. Fits of the Box-Lucas equation provided *SLP* values of 21 W/g and 34 W/g for CA- and PAA-coated samples, respectively. On the other hand, X-ray photoelectron spectroscopy analysis points to the catalytically active centers Fe^2+^/Fe^3+^ and Co^2+^/Co^3+^ on the particle surface, suggesting possible applications of the samples as heterogeneous self-heating catalysts in advanced oxidation processes under an AC magnetic field.

## 1. Introduction

When magnetic nanoparticles (MNPs) are subjected to an alternating (AC) magnetic field, they absorb the energy from the field and convert it into heat, leading to a temperature increase in the system. Consequently, they are suitable for various applications, including cancer treatment through magnetic hyperthermia (MH) [1,2]. Additionally, MNPs have been employed as heat generators for magnetically activable catalysis in the degradation of organic pollutants [3,4], as the reaction temperature plays a crucial role in the degradation efficiency. Also, MNPs assist in the generation of free radicals in heterogeneous catalysis processes [5,6].

MNPs, such as magnetite (Fe_3_O_4_), maghemite (γ-Fe_2_O_3_), and other spinel ferrites, are commonly used as heating agents in MH. Ferrites possessing the spinel structure are characterized by the general formula MFe_2_O_4_. Within the spinel unit cell, oxygen ions exhibit close packing alongside M^2+^ and Fe^3+^ ions, which are distributed between the tetrahedral (*8a*) and octahedral (*16d*) interstitial sites in the *Fd*$\bar{3}$*m* space group. While most ferrites with a spinel structure stick to the (M,Fe)_3_O_4_ stoichiometry, with a cation/anion ratio of 3:4, deviations from this stoichiometry are possible, particularly in nanoscale ferrites [7].

Various methods can be used to prepare these nanoparticles, with the co-precipitation technique being the most widely used and efficient pathway for achieving highly efficient synthesis. However, it comes with limitations related to wide size distribution and poor control over nanoparticle shape [8]. Numerous literature data highlight the impact of cation/anion doping or substitution on the properties of magnetic nanoparticles. The substitution of iron in the spinel structure of Fe_3_O_4_ with *3d* or *4f* ions through partial cation substitution opens up possibilities for enhancing magnetic and other physical properties, including heating efficiency in the AC magnetic field. Additionally, the physical properties of parent compounds can be altered by incorporation of polyvalent cations, such as Mn and Co. This approach often results in deviations from the ideal stoichiometry and the emergence of vacancies [7]. In spinel compounds, similar to iron, Co is predominantly found in +2 and +3 valence states. The introduction of doping elements changes the overall magnetic moment of nanostructures, depending not only on the values of the magnetic moments of substituent/dopant ions but also on their distribution in two cation non-equivalent crystallographic sites, *8a* (tetrahedral symmetry) and *16d* (octahedral symmetry). Superexchange interactions, which dominate as the primary type of magnetic interactions, particularly between magnetic ions in the *8a* and *16d* sites, are determined by the distribution of cations. In nanostructures, cation distribution is often imbalanced, and ion distribution frequently deviates from their energetically preferred states observed in their bulk counterparts [7].

In biomedical applications of MH, magnetic nanoparticles need to be in colloidal form, with good colloidal stability under physiological conditions, and possessing a uniform particle size distribution. To achieve colloidal stability, biocompatibility, and to prevent nanoparticle aggregation, MNPs are often coated with various organic and inorganic ligands [8]. Commonly used surface modifiers include (3-Aminopropyl)triethoxysilane (APTES), dimercapto succinic acid (DMSA), polyethylene glycol (PEG), citric acid (CA), human serum albumin (HSA), poly(lactic-co-glycolic acid) (PLGA), SiO_2_, and others [9,10,11]. It is noteworthy that, when MNPs are applied in vivo MH applications, the coating influences the formation of protein coronas in the biological milieu [12], impacting intracellular trafficking and degradation of MNPs [9]. However, the impact of coating materials on *SLP* has been studied to a limited extent. Coating MNPs with different molecules, such as PEG with varying molecular weights, affects the hydrodynamic diameters of particles. Consequently, this influences the Brownian contribution to *SLP*, as well as thermal conductivity, dispersibility, and inter-particle interactions [8,10,13].

Previous studies have shown that magnetite-based nanoparticles with variable cation valency are the most promising material to be used as heterogeneous catalysts for various organic reactions [3,14]. Wastewater treatments in heterogeneous Fenton and Fenton-like systems utilizing advanced oxidation processes (AOPs) in AC magnetic fields have been extensively studied over the past two decades. They were mainly based on the generation of free radicals ^•^HO and HOO^•^ in the reaction between H_2_O_2_ and active centers Fe(II)/Fe(III) [3,5]. Additionally, magnetic nanoparticles heating in the AC magnetic field enhances the catalytic process. Accordingly, MNPs acting as nanocatalysts should have both a large number of active sites on the surface of the particles and high hyperthermic efficiency. In this research, we studied the surface charge of (Co,Fe)_3_O_4_ nanoparticles by utilizing X-ray photoelectron spectroscopy (XPS) to determine possible ion oxidation states. It is worth mentioning the recent article by R. M. Sarimov et al. which provides a comprehensive analysis of the impact of low-frequency magnetic fields (EMFs) on the generation of reactive oxygen species (ROS) in biological systems [15]. There are several theoretical mechanisms explaining how magnetic fields affect living systems, including formation of spin-correlated radical pairs. EMFs have the potential to generate ROS-like singlet oxygen by inducing the formation of spin-correlated radical pairs in organisms [15]. These radicals can modulate biochemical reactions which play crucial roles in various physiological processes. The interaction between EMFs and biological systems, particularly through ROS generation, highlights the intricate mechanisms underlying magnetobiological effects [15].

In brief, we examined two distinct cobalt-substituted magnetite nanoparticles to assess the impact of cobalt concentration on the physical, chemical, and heating properties of (Co,Fe)_3_O_4_ nanoparticles. Additionally, we investigated the influence of two different types of surface modifiers on the *SLP* values. The prepared samples underwent comprehensive analysis using various and complementary experimental methods, including XRPD, TEM, ICP-OES, ATR-FTIR, SQUID magnetometry, DLS, zeta potential measurements and *SLP* measurements.

## 2. Experimental

### 2.1. Sample Preparation and Coating

#### 2.1.1. Reagents

All reagents were used as obtained and without any further purification treatment. Iron(III) chloride hexahydrate (FeCl_3_∙6H_2_O, reagent grade, ≥98%), ammonium iron(II) sulfate ((NH_4_)_2_Fe(SO_4_)_2_∙6H_2_O, ACS reagent, 99%), cobalt chloride hexahydrate (CoCl_2_∙6H_2_O, ACS reagent, 98%), sodium hydroxide (NaOH, ACS reagent, ≥97.0%, pellets), citric acid (C_6_H_8_O_7_, ACS reagent, ≥99.5%), poly(acrylic acid) (average *M_v_* ~1800), potassium hydroxide (KOH, ACS reagent, ≥85%, pellets) and nitric acid (HNO_3_ ACS reagent, 70%) were all supplied by Sigma-Aldrich, St. Louis, MO, USA. In all experiments, ultrapure Milli-Q water was used (Merck Millipore, Burlington, MA, USA).

#### 2.1.2. Synthesis of Co-Doped Magnetite

The samples were prepared using a co-precipitation method based on the recipe of Qu et al. [16], with some specific modifications. Notably, in this procedure, Fe(III) ions were not reduced in situ. Cobalt was introduced to replace 5 at. % and 10 at. % of Fe(II) ions in the parent magnetite compound. To synthesize the sample with the chemical composition Co_0.05_Fe_2.95_O_4_ (S1), 0.004 M FeCl_3_·6H_2_O, 0.0019 M (NH_4_)_2_Fe(SO_4_)_2_·6H_2_O, and 0.1 mM CoCl_2_·6H_2_O were dissolved in a specific amount of distilled water. Simultaneously, 13.3 mM of NaOH was added to a three-neck flask, and the mixture of metal ions was gradually added to the base dropwise over the duration of 25 min. After that, the system was heated to 80 °C in a water bath and stirred vigorously for 2 h using a mechanical stirrer under a blanket of N_2_ introduced to the system. For the synthesis of the second composition, Co_0.1_Fe_2.9_O_4_ (S2), the same procedure was repeated, but the amount of cobalt chloride hexahydrate and iron(III) chloride hexahydrate was changed accordingly. After the synthesis, the supernatant was removed, the precipitated solid product was washed with a mixture of ethanol and acetone and separated magnetically. Finally, the nanoparticles were dispersed in 15 mL of MilliQ water and stored as stable solutions at pH~3 and 4 °C in a refrigerator. To enhance their potential for biological applications and to improve colloidal stability at pH 7, the synthesized nanoparticles were coated with citric (CA) acid and poly(acrylic acid) (PAA).

#### 2.1.3. The Surface Modification of Nanoparticles

Prior to the coating process, preliminary magnetic hyperthermia tests were performed, and S1 was selected for surface modification due to its superior heat generation ability. The CA coating has been performed similarly to that described in our recent work [10]. In summary, 10 mL of the S1 (concentration 3.4 mg/mL) was mixed with 24 mL of 0.1 M CA in a single-necked flask and stirred magnetically at 80 °C for 30 min. Subsequently, the mixture was allowed to cool to room temperature (RT) and left to precipitate for 2 h. Afterward, 24 mL of the supernatant was carefully removed, and the residue was transferred to a 10 mL Float-A-Lyzer^®^ (100 kDa) dialysis membrane and dialyzed overnight in distilled water. Finally, the pH was first adjusted to 11 using 0.2 M KOH and then to 7 with the addition of 0.05 M HNO_3_. The sample coated with citrates will be further referred to as S1@CA.

PAA-coated S1 nanoparticles were prepared by mixing 30 mg of S1 magnetic nanoparticles with 30 mg of PAA oligomer in 10 mL of distilled water at 60 °C. The mixture was stirred at 400 rpm for 20 min. The S1@PAA was purified by centrifugation, washing, and redispersion cycles and then dialyzed against deionized water [17,18].

### 2.2. Characterization Techniques

The inductively coupled plasma optical emission spectroscopy (ICP-OES) (Thermo Fisher Scientific, iCAP some Duo ICP, Cambridge, UK) was employed to determine the iron and cobalt content in colloidal dispersions. Quantification was carried out following total acid digestion, and measurements were taken at emission wavelengths of 259.83 nm (for Fe) and 228.615 nm (for Co).

The crystal structure of Co-substituted magnetite was determined by analyzing X-ray powder diffraction (XRPD) data. The measurements were performed on dried powders using a high-resolution Smart Lab^®^ diffractometer (Rigaku, Tokyo, Japan), equipped with a Cu Kα radiation source (λ = 1.5406 Å) under a voltage of 40 kV and a 30 mA current. The data collection for the patterns was performed in the 10–110° 2θ range. The X-ray diffraction data were collected at a scan rate of 0.5° per minute with a step size of 0.02° during the scan. The phase identification of the synthesized materials was carried out using the Halder–Wagner method incorporated in the PDXL2-integrated X-ray powder diffraction software (Version 2.8.40, Rigaku Corporation, Tokyo, Japan).

The microstructure and morphology of the synthesized samples were examined using a transmission electron microscope (TEM) JEOL-JEM 1010 (JEOL, Tokyo, Japan), operating at 100 kV. The sample preparation involved placing a drop of particles suspended in water onto a carbon-coated copper grid and allowing it to dry at RT for TEM observations. The TEM images were manually analyzed using ImageJ software. The mean particle size and distribution were determined by measuring the largest internal dimension of 150 nanoparticles per sample. Subsequently, the data were fitted to a log-normal function to derive the mean size, standard deviation (σ*_TEM_*), and the index of polydispersity (*PdI*), which reflects the measurement’s absolute error.

The presence of organic molecules acting as capping agents on the particle surfaces was examined using Fourier transform-infrared spectroscopy (FT-IR) within the 4000–400 cm^−1^ range. The analysis was performed on a Nicolet iS50 FT-IR spectrophotometer equipped with a Smart iTR attenuated total reflectance (ATR) sampling accessory. The powder samples were affixed onto a diamond plate using a swivel press, ensuring optimal contact between the sample and the crystal. The background spectra were measured on a clean, dry diamond crystal and automatically removed by OMNIC™ Specta Software (Thermo Fisher Scientific, Waltham, MA, USA).

Colloidal properties of materials were analyzed using a Zetasizer Nano ZS90 (Malvern Panalytical, Malvern, UK). Zeta potentials of 0.1 mg/mL suspensions containing bare and coated S1 nanoparticles dispersed in deionized water were measured at 25 ± 0.1 °C in disposable zeta cells (DTS 1070). The initial stock suspensions were diluted to 0.1 mg/mL. Before measurements, the samples were equilibrated for 10 min. The hydrodynamic diameters were determined via dynamic light scattering (DLS) using a NanoZS90 instrument equipped with 4 mW He–Ne laser source (λ = 633 nm).

To assess the magnetic properties, approximately 10 mg of bare S1 and S2 powders were packed into polycarbonate capsules and secured with cotton wool. Hysteresis loops were measured using a SQUID magnetometer (MPMS-5, Quantum Design Inc, San Diego, California, USA) at 300 K, applying fields up to 30 kOe at a field change rate of 3 kOe per minute, subsequent to sample saturation in a 30 kOe field. Additionally, information regarding the blocking temperature (*T_B_*) was obtained by analyzing magnetization data within the temperature range of 5–300 K, employing the zero-field cooled (ZFC) and field cooled (FC) regimes using a DC magnetic field of 100 Oe.

The saturation magnetization (*Ms*), average particle size (*d_avg_*) and standard deviation *σ* were calculated through fitting of the weighted Langevin function, $\int g_{n}\left(d,s,D\right)L\left(H\right)dD$, to the acquired hysteresis data [19]. In the above formula, $g_{n}\left(d,s,D\right)$ represents log-normal distribution (Equation (1)),

(1) $$g_{n}\left(\bar{d},\sigma ,D\right)=\frac{1}{\sqrt{2\pi }sD/d}exp\;\left[-\frac{{ln\;\left[D/d\right]}^{2}}{2s^{2}}\right],$$

where *D* is particle diameter, and *d* and *s* represent distribution parameters. $L\left(H\right)$ is the Langevin function,

(2) $$L\left(H\right)=Ms\;\left(Coth\left(\mu_{0}H\;\mu /kT\right)-{\left(Coth\left(\mu_{0}H\;\mu /kT\right)\right)}^{-1}\right),$$

where *μ* represents magnetic moment of the particle, *k* Boltzmann constant, and *T* measurement temperature (Equation (2)). The particles were approximated by perfect spheres ($V=\pi D^{3}/6$) with homogenous magnetization throughout the particle (μ=Ms V).

The Box-Lucas equation is derived for the case when losses to the environment depend linearly on temperature difference between sample and environment, and reads as follows (Equation (3)):

(3) $$T=T_{0}+\frac{m\;SLP}{L}\left(1-e^{-\frac{L\;t}{C}}\right),$$

where *T*_0_ represents the temperature of the environment, *m* the mass of the particles in dispersion, *C* water heat capacity, and *L* coefficient of heat losses to the environment.

Calorimetric measurements performed using a commercial AC hyperthermia device (model DM 100 nB nanoScale Biomagnetic, Zaragoza, Spain). Heating curves were recorded under a 252 kHz frequency with a 200 Oe AC field. A total volume of 1 mL containing co-doped magnetite colloidal dispersions in water concentration of 3–5 mg mL^−1^ was introduced into a measuring cuvette. The temperature increase was continuously monitored over 5 min using an optical fiber system.

X-ray photoelectron spectroscopy (XPS) measurements were conducted using the AXIS Supra electron-spectrometer from Kratos Analitycal Ltd. (Stretford, UK), featuring an analysis chamber with a base pressure better than 10^−9^ mbar. For the measurements, the AlKα non-monochromated X-ray source (1486.6 eV) was utilized, employing a 1 mm beam spot. No energy calibration was performed because a charge compensator was used. The instrumental resolution is better than 0.5 eV, measured as the full width at half maximum (FWHM) of the Ag3d_5/2_ photoelectron peak. The spectra are recorded at the total instrumental resolution of ±0.1 eV. CasaXPS software (Casa Software Ltd., Devon, UK) was employed for data analysis. The spectral processing involved the subtraction of a Shirley-type background [20]. The peak positions and areas are evaluated by a symmetrical Gaussian–Lorentzian curve fitting based on the standard spectra of cobalt and iron for different oxidation states. Relative concentrations of different chemical species were derived by normalizing peak areas to their photoionization cross-sections, calculated using the Scofield method [21].

## 3. Results and Discussion

### 3.1. Formation of Nanoparticles and (Micro)Structural Analysis of Co-Doped Magnetite

ICP-OES elemental analysis has been performed to investigate the substitution of iron ions with cobalt ions in the structure of magnetite (Fe_3_O_4_). The results indicated a slightly lower concentration of cobalt compared to the targeted values (Table 1).

**Figure 1:**
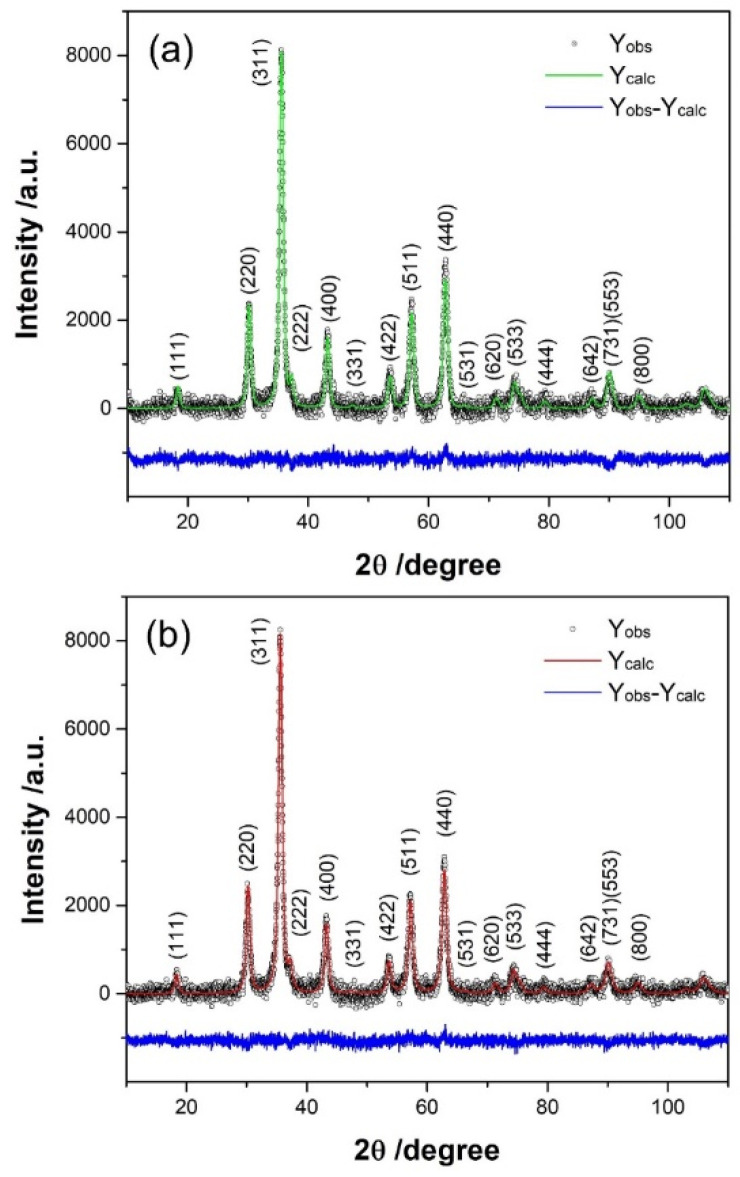
The refined diffraction patterns of (**a**) Co_0.047_Fe_2.953_O_4_ (S1) and (**b**) Co_0.086_Fe_2.914_O_4_ (S2). The bottom line indicates the difference between data and calculated pattern.

The crystal structures of the samples were analyzed using X-ray powder diffraction data. In the obtained diffraction patterns, all reflections were successfully indexed in the space group *Fd*$\bar{3}$*m*. These patterns revealed a pure spinel, without other crystal phases or impurities. X-ray diffraction data were utilized to deduce both the crystal structure and crystallite size of the samples. Rietveld refinement analyses of XRPD data were performed using the integrated X-ray powder diffraction software PDXL2. Figure 1 illustrates the results of the Rietveld refinement procedure, confirming excellent agreement between the structural model and experimental data.

The determined size of crystallites for both investigated samples was approximately 10 nm (see Table 1). Refined values of lattice parameters showed slight differences, attributed to variations in ionic radii and cation distribution between two non-equivalent crystallographic sites in the spinel structure: tetrahedral (*8a*) and octahedral (*16d*) (see Reference [7]). However, a detailed analysis of lattice parameter changes requires knowledge of cation distribution. Varying the occupancy numbers during refinement to determine ion distribution proved insensitive, primarily due to the similarity in the scattering factor of cobalt and iron.

**Figure 2:**
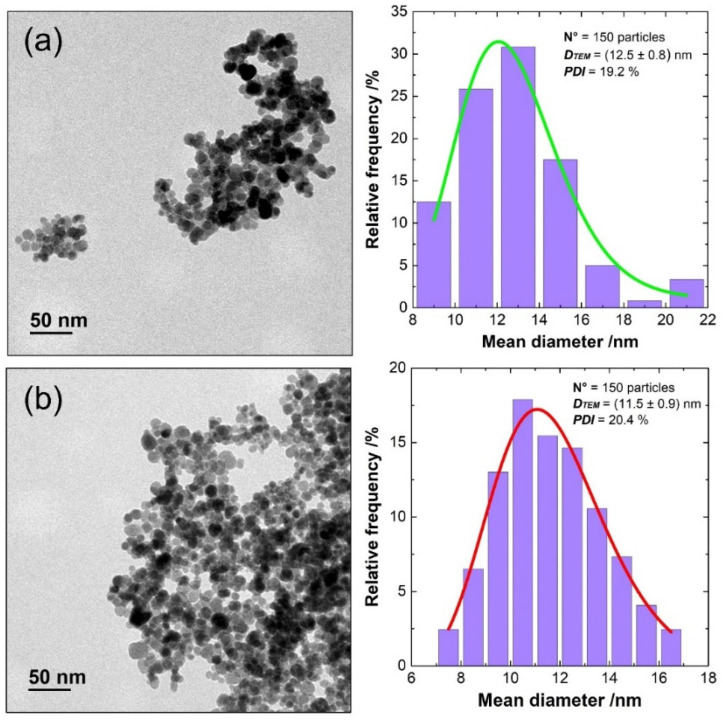
TEM micrographs of (**a**) Co_0.047_Fe_2.953_O_4_ (S1) and (**b**) Co_0.086_Fe_2.914_O_4_ (S2). The corresponding statistical particles’ size distribution has been given next to the figures.

The transmission electron microscopy (TEM) was used to investigate the morphology and size of nanoparticles and correlate them with chemical composition (cobalt concentration). As observed, these particles exhibit a quasi-spherical shape. A statistical analysis determined the mean particle size and their distributions, showcased in Figure 2 and summarized in Table 1. The differences in average particle sizes of two samples fall within the margin of error. The obtained values for crystallite size and particle size are in agreement, which is an indication that the nanoparticles are composed mainly of one crystallite.

### 3.2. Coating and Colloidal Properties of Co-Doped Magnetite Nanoparticles

Co-substituted magnetic nanoparticles were coated with two organic ligands that stabilize colloidal dispersions electrostatically, utilizing unbound carboxyl groups on their surface. For this purpose, molecules with different molecular weights were chosen: CA as a small molecule and PAA as a relatively large molecule.

**Figure 3:**
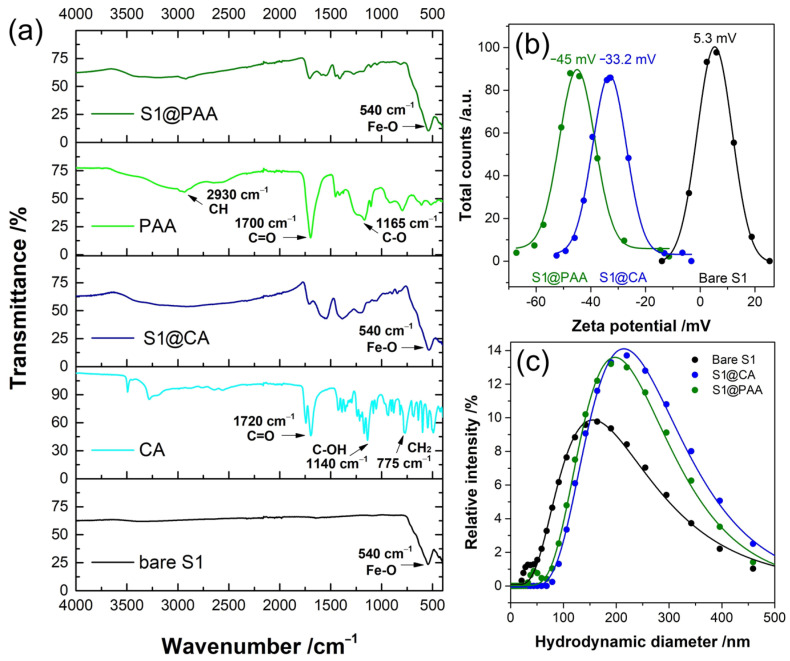
(**a**) ATR-FTIR spectra of bare S1, CA- and PAA-coated S1; (**b**) zeta potential measurements and (**c**) hydrodynamic diameters of bare and coated S1 nanoparticles.

ATR-FTIR spectra of S1, selected for surface modification, before and after functionalization with CA and PAA are shown in Figure 3a. The ATR-FTIR spectra of bare S1 (black line) revealed a signature stretching Fe–O vibration at ~540 cm^−1^, in line with the literature data [22]. The ATR-FTIR study was primarily conducted to confirm the presence of CA and PAA on the S1 surface following the functionalization processes (S1@CA and S1@PAA). The 1720 cm^−1^ spectral peak of CA arises from the symmetric C=O stretching vibration of the carboxylic group. This peak shifts to a lower value band, approximately 1555 cm^−1^, for the carboxylic group (R-COOH) of S1@CA, attributed to the chemisorption of carboxylate citrate ions. The peak at 1384 cm^−1^ corresponds to the asymmetric stretching of C–O from the −COOH group. Additionally, the strong vibration band observed at 540 cm^−1^ is attributed to the Fe–O stretching vibrational mode of Co-doped magnetite, which is absent in the CA spectra [22].

The ATR-FTIR spectra depicted the comparison before and after functionalization with PAA in the upper section of Figure 3 (green line). In the PAA spectra, notable peaks at 2930 cm^−1^, 1455 cm^−1^ and 1412 cm^−1^ correspond to ${-\mathrm{CH}}_{2}^{-}$ (stretching and bending), –COO (stretching in –COOH), and C-O (stretching in –COOH), respectively. Both spectra of PAA exhibit a prominent C=O band at around 1700 cm^−1^, indicative of carboxylate acid species. In the S1@PAA spectra, the C=O band shifts from 1695 cm^−1^ to 1706 cm^−1^, suggesting potential ester linkage formation. The –CH_2_ scissoring vibrations band at 1454 cm^−1^ is present in the PAA sample and remains consistent in both spectra. Similar to the citrate coating, the vibrational band at 540 cm^−1^ is observed in the S1@PAA sample but is notably absent in the PAA spectra.

The variation in particle charge occurs due to the deprotonation and protonation of hydroxyl groups on the particle surfaces. In an acidic environment, nanoparticles carry a positive charge due to the protonation of these hydroxyl groups. However, at alkaline pH values, the particle surfaces acquire a negative charge as a result of the dissociation of the –OH groups, forming deprotonated hydroxyl groups as Fe–O^–^.

Zeta potential measurements at the physiological pH are shown in Figure 3b. The zeta potential results indicate the successful implementation of coating protocols of the bare S1 nanoparticles. In contrast to the zeta potential of bare S1 (5.3 mV), both S1@CA and S1@PAA nanoparticles exhibit negative surface charges of −33.2 mV and −45.2 mV, respectively. The change in zeta potentials contributes to increased repulsion among nanoparticles owing to Coulombic forces, thereby facilitating the electrostatic stabilization of the nanoparticle suspension. These results suggest the existence of unreacted carboxyl groups on the nanoparticles’ surface and the robust coordination of certain carboxylic groups from CA and PAA with iron and cobalt cations.

The DLS method determined the hydrodynamic diameters of the nanoparticle aqueous suspension at pH 7, as depicted in Figure 3c. The intensity distribution on the graphs reveals the size distribution of particles within the suspensions. In the uncoated S1 suspension, a dominant peak at around 30 nm suggests the presence of a large population of hydrated, individual nanoparticles. Additionally, a smaller peak at approximately 170 nm indicates a minor presence of agglomerates. A similar trend is observed for S1@PAA, with primary particles centered around 50 nm and a smaller number of agglomerates at 210 nm. However, the S1@CA suspension exhibits a distinct characteristic. The absence of a peak at smaller particle sizes suggests a limited population of individual nanoparticles. This observation may indicate a higher degree of agglomeration within the S1@CA suspension, leading to a larger average particle size. The observed *D_TEM_* and *D_H_* difference stems from the fact that the *D_H_* value represents particle size in their hydrated state, including polymer chains extending from the particle surface into the aqueous phase. This incorporation contributes to an augmented overall particle size. The DLS results not only validate the successful binding of CA and PAA but also indicate potential nanoparticle agglomeration as a possible explanation for these differences.

### 3.3. Magnetic Properties and Heating Efficacy of Co-Doped Magnetite

Isothermal magnetization was measured for both S1 and S2 samples in the magnetic field ranging from −30 kOe to 30 kOe at RT. Both samples show negligible coercivity and remanent magnetization, 6 Oe and 0.5 emu/g, for sample S1, and 20 Oe and 1.4 emu/g, for sample S2. The increase in remanence and coercivity with Co content could be attributed to an increase in magnetocrystalline anisotropy [23,24], making the S2 sample slightly magnetically harder than S1.

**Figure 4:**
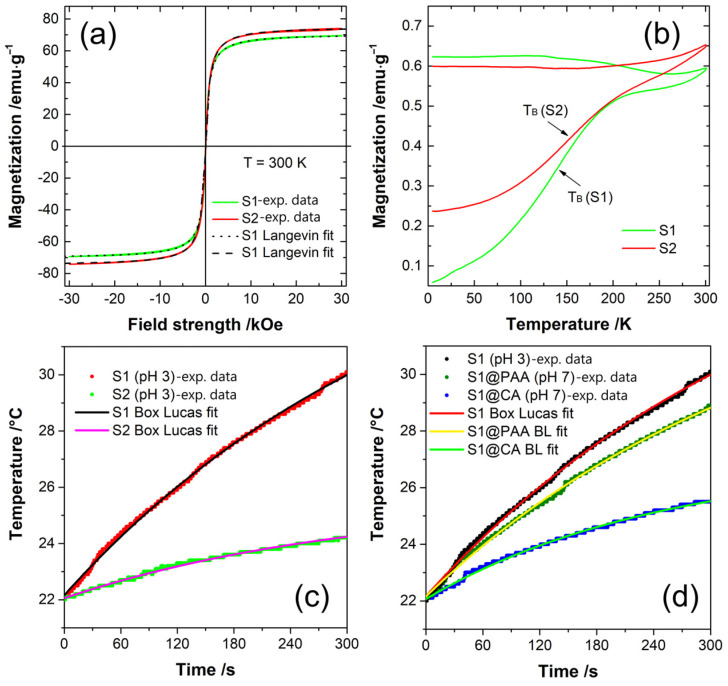
(**a**) Magnetization vs. magnetic field of S1 and S2 nanoparticles at 300 K; (**b**) ZFC/FC magnetization vs. temperature curves measured under an applied magnetic field of *H* = 100 Oe; (**c**) heating curves of bare S1 and S2 at pH 3 and (**d**) heating curves of S1, CA- and PAA-coated S1 under *H_AC_* = 200 Oe and *f* = 252 kHz.

The saturation magnetization *Ms*, average particle size *d_avg_* and standard deviation *σ* were calculated through fitting of the weighted Langevin function (Equation (2)) to the acquired hysteresis data (Figure 4a). The fitting gave us the saturation magnetization values of 71 emu g^−1^ and 75 emu g^−1^ for samples S1 and S2, respectively (Table 2). The acquired values agree with the literature values commonly found for nano-sized magnetite and cobalt ferrite [25]. The difference in *Ms* for samples with different Co content is already reported by several authors [16,23,24]. It could be attributed to the different content of maghemite in the samples, which has a lower saturation magnetization value than magnetite, or to the random placement of Co^2+^ ions in both A- and B-site lattice positions leading to higher uncompensated B-sublattice magnetization in the sample with higher Co content [26,27].

The average diameter values acquired from the fits agree with the values deduced from the analysis of the XRPD results (Table 1). The given values can be regarded as the average size of the magnetically ordered particle cores, without the disordered magnetically “dead” shell [28]. This does not exclude the possibility of an existence of a spin-disordered layer at the particle surface.

ZFC/FC magnetization measurements were conducted in the 100 Oe field, in the temperature range from 5 K to 300 K (Figure 4b). Flat FC curves show the presence of significant interparticle interactions in both samples [29]. This is most likely due to particle agglomeration in measured powders. The *T_B_* deduced from the maximum of the first derivative of the ZFC-FC difference, are 139 K and 153 K for the S1 and S2 sample, respectively. Similarly, to increase the coercivity and remanence, the increase in *T_B_* could be attributed to the increase in magnetocrystalline anisotropy due to incorporation of Co ions into the lattice [23].

The efficiency of heat generation by MNPs is quantified by the Specific Loss Power (*SLP*), often determined through calorimetric heating measurements [30]. During the measurement, the samples were subjected to an AC magnetic field with an amplitude of 200 Oe and frequency of 252 kHz. The *SLP* can be calculated by fitting the Box-Lucas equation to the acquired temperature curves [31].

The Box-Lucas equation can be used for the description of temperature curves where the temperature difference between the sample and environment does not surpass 10–15 °C [32] (Equation (3)). Figure 4c shows measured temperature curves of dispersions of samples S1 and S2. The flocculation and sedimentation of particles during the measurement was prevented by lowering the pH of dispersions to 3. The fits of the Box-Lucas equations to temperature curves gave *SLP* values of 46 W/g and 23 W/g, for S1 and S2, respectively.

Sample S1, due to its better heating efficacy, was chosen for further investigation of the coating effect. Temperature curves of the bare S1, as well as CA- and PAA-coated are shown in Figure 4d. Fits of the Box-Lucas equation gave *SLP* values of 21 W/g and 34 W/g for the CA- and PAA-coated samples, respectively. Results of the hydrodynamic size measurements (Figure 3c) hint that instead of separate particles, small agglomerates were coated with CA and PAA. If this is the case, the lower *SLP* of the coated samples could be attributed to the presence of interparticle interactions and inhibition of particles’ Brown relaxations [33].

### 3.4. XPS Analysis

**Figure 5:**
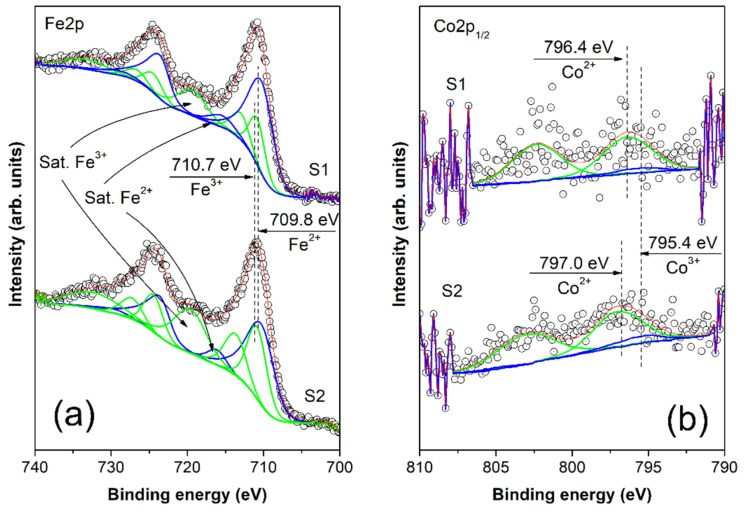
High-resolution XPS spectra of (**a**) Fe2p and (**b**) Co2p_1/2_ core levels measured for nanoparticles of Co_0.047_Fe_2.953_O_4_ (S1) and Co_0.086_Fe_2.914_O_4_ (S2). The open circles represent the experimental data, while the oxidation states retrieved from the curve-fitting procedure are depicted with solid lines—blue for 3+ and green for 2+. The red line is employed for the envelope.

X-ray photoelectron spectroscopy was employed to probe the oxidation states and atomic concentrations of surface elements within the nanoparticles. Survey spectra data unveils the core level peaks, encompassing iron (Fe2p), cobalt (Co2p), oxygen (O1s), and carbon (C1s), constituting the nanoparticles. Additionally, minor residuals from the preparation procedure, such as chlorine (Cl2p core level), along with barely detectable traces of sodium (Na1s core level), are also observed. More detailed information can be gained from the high-resolution XP spectra shown in Figure 5. In Figure 5a, the Fe2p core level energy range is depicted. A meticulous examination of the curve’s line shape, coupled with a comparative analysis against standard spectra measured for Fe^2+^ and Fe^3+^ oxidation states, suggests a combination of these two oxidation states for both samples, S1 and S2 [34,35]. Employing a curve fitting procedure based on parametrized standards, we utilized FeO (standard for Fe^2+^) and Fe_2_O_3_ (standard for Fe^3+^). The standards included peak positions, satellites, and peak area ratios for precise analysis. For Fe^2+^, the FeO standard featured a Fe2p_3/2_ peak binding energy (BE) of 709.8 eV, a satellite in the energy range of 714.5–715.5 eV, and multiplet splitting Δ = Fe2p_3/2_ − Fe2p_1/2_ = 13.5 eV, with Area(Fe2p_1/2_)/Area(Fe2p_3/2_) maintained at 1/2. Meanwhile, for Fe^3+^, the Fe_2_O_3_ standard exhibited a Fe2p_3/2_ peak BE of 710.7 eV, a satellite in the energy range of 718.5–719.5 eV, and Δ = Fe2p_3/2_ − Fe2p_1/2_ = 13.6 eV, with the same area ratio of 1/2. This enabled the determination of the Fe^2+^/Fe^3+^ ratio, yielding values of 1.37 for sample S1 and 0.68 for sample S2, as detailed in Table 2. The obtained ratio represents a deviation from the expected value in the core of nanoparticles, where for stoichiometric Fe_3_O_4_, the Fe^2+^/Fe^3+^ ratio is 0.5. It is known that in nanoparticles, due to small dimensions and a large surface-to-volume ratio, a relatively large number of atoms are located on the surface, where chemical bonds are broken, translational symmetry is lost, and coordination is lower. Significant deviation of the Fe^2+^/Fe^3+^ ratio on the surface from the expected ratio for core nanoparticles can be explained by the mentioned surface effects. The difference in surface charge between S1 and S2 is more likely a result of poorly controlled synthesis methods rather than the influence of cobalt concentration. The presence of catalytically active centers of Fe^2+^/Fe^3+^ on the particle surface is important for heterogeneous Fenton and Fenton-like reactions in the potential application of these nanoparticles for the degradation of organic pollutants [5]. It has been shown that Fe^2+^ and/or Fe^3+^ surface ions can react with hydrogen peroxide to produce highly oxidative species for the degradation of organic molecules. Additionally, magnetic iron oxide-based particles in an alternating magnetic field can become “self-heating” catalysts, enhancing degradation processes [36].

In Figure 5b, we present the energy range of the Co2p_1/2_ core level, with a pertinent note that the analysis of Co2p_3/2_ is deemed irrelevant due to the overlapping Auger peak FeLMM of iron. It is crucial to highlight that the concentration of cobalt in both samples, S1 and S2, is less than 0.5 at. %, introducing challenges in the form of low intensity and noisy spectra, thus complicating the analysis and reducing accuracy. We have applied a similar curve fitting procedure for the Co2p_1/2_ peak as the procedure for Fe2p above. This procedure utilized parametrized standard spectra for Co^2+^ and Co^3+^ and it has been successfully applied in Refs. [37,38,39].

The Co^2+^ standard featured a peak in the range of 796.5–797.5 eV, accompanied by a relative intensive 3d→4s “shake-up” satellite peak with the same peak area and positioned 6.0–6.5 eV apart from the 2p_1/2_ core level BE. On the other hand, the Co^3+^ standard exhibited a 2p_1/2_ core level BE at 795.0–795.5 eV, with a low intensive 3d→4s “shake-up” satellite with a peak area constituting about a quarter of the 2p_1/2_ core level peak area and positioned approximately 9.0–9.5 eV higher in BE. The outcome of the curve fitting procedure revealed that the minor amount of cobalt incorporated into the iron-oxide structure predominantly exists in the Co^2+^ oxidation state. Upon closer inspection of the peak shape, it is suggested that less than 10% of the total cobalt could potentially be in the Co^3+^ oxidation state for both samples S1 and S2 (refer to Table 3).

Furthermore, an intriguing observation is the cobalt ions’ concentration on the surface, which is approximately 40 times lower than that of iron ions. It is useful to mention the results of the study of Tatarchuk et al., where Co-ferrite was tested as a nanocatalyst in a variable magnetic field [40]. The study showed that the induction heating of the cobalt ferrite catalyst accelerates the reaction rate by up to two times. The high catalytic activity of cobalt ferrite is attributed to iron cations, which reversibly changes their oxidation state. Consequently, highly reactive hydroxyl radicals are formed, capable of non-selective oxidation of organic pollutants. Cobalt Co^2+^ ions react with H_2_O_2_ in a manner similar to Fe^2+^, producing HO^•^ radicals in a Fenton-like process [40]. This highlights the potential of magnetic nanoparticles (cobalt ferrite) as nanocatalysts for efficient degradation of organic pollutants, with the added advantage of induction heating in a variable magnetic field to enhance the catalytic activity. Consequently, Co^2+^/Co^3+^ on the surface of studied nanoparticles acts as an active center for redox processes in catalytic applications of self-heating nanoparticles.

## 4. Conclusions

Two samples with similar chemical compositions, Co_0.047_Fe_2.953_O_4_ and Co_0.086_Fe_2.914_O_4_, were synthesized to assess how a slight change in chemical composition affects their physical and chemical properties. The *T_B_* were 139 K and 153 K for samples S1 and S2, respectively. Similar to the increase in coercivity and remanence, which were 6 Oe and 0.5 emu/g for sample S1, and 20 Oe and 1.4 emu/g for sample S2, the increase in *T_B_* could be attributed to an increase in magnetocrystalline anisotropy due to the higher concentration of Co ions. Heating efficiency tests showed that sample S1 (*SLP* was 46 W/g) exhibited higher heating efficiency compared to S2 (*SLP* was 23 W/g). Coating Co-substituted Fe_3_O_4_ nanoparticles resulted in stable colloids, the properties of which depend on the type of compound used for coating. Consequently, by selecting the coating compound, colloidal properties can be tailored for specific applications, such as magnetic hyperthermia in cancer therapy. Surface modification of nanoparticles with CA and PAA affected the *SLP* values due to changes in the Brownian contribution and potential presence of interparticle interactions and agglomeration. Calculated *SLP* values were 21 W/g and 34 W/g for the CA- and PAA-coated S1 samples, respectively. Furthermore, as heat sources, the samples have potential applications in catalytic processes. X-ray photoelectron spectroscopy (XPS) revealed the presence of cobalt and iron ions on the particles’ surfaces in two different oxidation states, indicating that the samples can facilitate redox processes on the surface and be used as heterogeneous catalysts. Surface concentrations of cobalt ions were significantly lower than iron ions, with Fe^2+^/Fe^3+^ ratios of 1.37 for sample S1 and 0.68 for sample S2. Samples under investigation in this study contain small amounts of cobalt. However, it is crucial to examine the possibility of cobalt leaching from its compounds found in the body for biomedical applications. Elevated levels of cobalt are known to lead to neurological, cardiovascular, and endocrine disorders [41]. Therefore, before conducting in vitro and in vivo tests for potential nanoparticle use as agents for magnetic hyperthermia, it is important to perform cytotoxicity assays and cobalt leaching tests.

## Figures and Tables

**Table 1:** Chemical composition of S1 and S2 according to stoichiometric ratio of starting compound (targeted) and actual composition determined by ICP-OES. Crystallite size (D_XRPD_), lattice parameter (a), average nanoparticle size (D_TEM_) and polydispersity index (σ_TEM_) are listed.

#	Chemical Composition (Targeted)	Chemical Composition (ICP-OES)	*D_XRPD_* (nm)	*a* (Å)	*D_TEM_* (nm)	*σ_TEM_* (nm)	*PdI* (%)
S1	Co_0.05_Fe_2.95_O_4_	Co_0.047_Fe_2.953_O_4_	10.0 (7)	8.3690 (8)	12.5 ± 0.8	0.2522	19.2
S2	Co_0.1_Fe_2.9_O_4_	Co_0.086_Fe_2.914_O_4_	9.5 (2)	8.3782 (8)	11.5 ± 0.9	0.2593	20.4

**Table 2:** Results of the weighted Langevin fit.

#	*Ms* (emu g^−1^)	*d_avg_* (nm)	*σ* (nm)
S1	71 *	9	3
S2	75	8	2

* The accompanying fitting standard deviations are of the order 10^−2^ or smaller. The values are rounded and errors are omitted to keep the table clear.

**Table 3:** Surface atomic concentration (at. %) of elements of Co_0.047_Fe_2.953_O_4_ (S1) and (b) Co_0.086_Fe_2.914_O_4_ (S2) nanoparticles.

#	Chemical Composition	C1s	O1s	Cl2p	Fe2p		Co2p	
S1	Co_0.047_Fe_2.953_O_4_	47.01	38.37	1.47	7.44	5.43	0.27	0.02
S2	Co_0.086_Fe_2.914_O_4_	31.41	45.92	2.51	7.94	11.74	0.43	0.05
	Ox. state				Fe^2+^	Fe^3+^	Co^2+^	Co^3+^

## Data Availability

The study did not report any data.
